# Non-Destructive Imaging on Synthesised Nanoparticles

**DOI:** 10.3390/ma14030613

**Published:** 2021-01-29

**Authors:** Kelvin Elphick, Akinobu Yamaguchi, Akira Otsuki, Neil Lonio Hayagan, Atsufumi Hirohata

**Affiliations:** 1 Department of Electronic Engineering, University of York, Heslington, York YO10 5DD, UK; kelvin.elphick@gmail.com; 2 Laboratory of Advance Science and Technology for Industry, University of Hyogo, Hyogo 678-1205, Japan; yamaguti@lasti.u-hyogo.ac.jp; 3 Ecole Nationale Supérieure de Géologie, GeoRessources UMR 7359 CNRS, University of Lorraine, 2 Rue du Doyen Marcel Roubault, BP 10162, 54505 Vandoeuvre-lès-Nancy, France; akira.otsuki@univ-lorraine.fr (A.O.); hayaganneil@gmail.com (N.L.H.); 4 Waste Science & Technology, Luleå University of Technology, SE 971 87 Luleå, Sweden; 5 Neutron Science Laboratory, The Institute for Solid State Physics, The University of Tokyo, Chiba 277-8581, Japan

**Keywords:** scanning electron microscopy, backscattered electrons, electron flight simulation, nanoparticles, synthesis

## Abstract

Our recently developed non-destructive imaging technique was applied for the characterisation of nanoparticles synthesised by X-ray radiolysis and the sol-gel method. The interfacial conditions between the nanoparticles and the substrates were observed by subtracting images taken by scanning electron microscopy at controlled electron acceleration voltages to allow backscattered electrons to be generated predominantly below and above the interfaces. The interfacial adhesion was found to be dependent on the solution pH used for the particle synthesis or particle suspension preparation, proving the change in the particle formation/deposition processes with pH as anticipated and agreed with the prediction based on the Derjaguin–Landau–Verwey–Overbeek (DLVO) theory. We found that our imaging technique was useful for the characterisation of interfaces hidden by nanoparticles to reveal the formation/deposition mechanism and can be extended to the other types of interfaces.

## 1. Introduction

Nanoparticles have been synthesised on metallic electrodes and (non-)conductive substrates. Their properties are known to be controlled by their interfacial structures governed by their formation processes. To date, these interfaces have been predominantly imaged by destructive methods, which can achieve nanometric resolution. As reported earlier [1], the highest resolution can be achieved by (scanning) transmission electron microscopy ((S)TEM) and atom probe imaging. These methods have been used commonly for the nano- to atomic-scale analysis of the junction interfaces. However, they require samples to be milled for electron transparency, introducing possible strain and defects during the sample preparation and hindering the direct correlations between the interfacial structures and electromagnetic properties. Electron beam-induced and -absorbed currents (EBIC and EBAC, respectively) has also been used, especially in semiconductor industries, but they are limited to transport properties with the most conductive layer with a sub-micron resolution.

On the other hand, our recently developed non-destructive imaging method can be performed by controlling the acceleration voltage in scanning electron microscopy (SEM) without modifying a sample and a device [2]. This method achieves a high in-plane resolution of a few nm without any additional requirements of sample preparation for imaging. By controlling the electron acceleration voltages in SEM, the penetration depth of the electron beam can be manipulated. The corresponding generation of secondary electrons (SEs) and backscattered electrons (BSEs) are generated within the electron plume introduced. Since SEs can be surface sensitive via following scattering processes within the specimen, BSEs are detected in this non-destructive imaging method using an energy filter. Recently, we have demonstrated in situ imaging capability under the current-voltage applications, allowing direct comparisons with the defects and the electrical transport properties [3]. Further, the combinations of spectroscopic and scattering/reflective chemical analysis allowed us to evaluate the origins of the defects, which is ideal as a quality assurance for nano-electronic industries. The defect details and the corresponding transport properties can be fed back to the processes of the device fabrication processes, improving the yields [4].

In this study, we applied our method to the characterisation of nanoparticles. We prepared two types of nanoparticles by X-ray radiolysis and the sol-gel method. By imaging these nanoparticles using our method, clear differences in their interfacial structures were found. They revealed the differences in their formation processes during the synthesis or particle suspension preparation, and confirmed the formation/dispersion models predicted depending on the solution pH used for the particle synthesis or particle suspension preparation. Hence, our imaging method can be highly useful for the understanding of the particle synthesis/dispersion processes and can be fed back to the process optimisation of nanoparticle systems.

## 2. Synthesis of Nanoparticles and Preparation of Nanoparticle Suspensions

### 2.1. X-ray Radiolysis

X-ray radiolysis was used to synthesise nanoparticles using beam line BL8S2 at the Aichi Synchrotron Radiation Center, Aichi Science & Technology Foundation. As detailed in our previous publication [5], a 100-mL aliquot of 0.37 mol/L (M) Cu(COOCH_3_)_2_ (FUJIFILM Wako Pure Chemical Corporation, Osaka, Wako 1st Grade, Japan) was prepared by diluting the stock solution and mixed with methanol with the volume ratio shown in Table 1. In total, 20 µL of these solutions were spread on Si/SiO_2_, *n*-Si, or Cu substrate, followed by the exposure of 5 min of synchrotron X-ray radiation to synthesise nanoparticles. X-ray irradiation can generate radicals from the radiolysis of liquids and secondary electron generation from substrates dipped in metallic liquid solution. There are some possible routes for the nucleation, ripping, growth, aggregation, and immobilisation of the particles onto the surface of substrate. In particular, near the substrate surface and the interface between the particles and the substrate, the nucleation, growth, and aggregation of these particles can be controlled by the X-ray irradiation significantly. Therefore, this investigation by non-destructive imaging is significantly worthwhile for the understanding of the physical and chemical mechanisms for the synthesis of particles. Simultaneously, this study can also provide the clue to control the synthesis and immobilisation of the particles.

### 2.2. Nanoparticle Synthesis by the Sol-Gel Method and Suspension Preparation

Monodispersed silica nanoparticles were prepared by using the method proposed by [6]. The average particle radius measured by using TEM images was 280 nm and used in the DLVO (Derjaguin–Landau–Verwey–Overbeek) potential calculation (see Section 3). The stock solution containing synthesised silica particles was washed several times to minimise the salt concentration prior to preparation of the desired silica particle suspensions for investigation with the desired chemical environments. Under the different conditions listed in Table 2, silica particle suspensions were prepared in aqueous salt solution (1 × 10^−2^ M KNO_3_, Sigma-Aldrich (St. Louis, MO, USA), and their pH was adjusted using HNO_3_ or KOH followed by conditioning the suspensions for 30 min. A tiny volume of each sample was pipetted and deposited on a standard SEM aluminium stub that was left in an oven at 50 °C for several hours to let the moisture content evaporate and firmly deposit particles on the stub by capillary forces with the residual moisture, followed by metallization of the stub for the sample conductivity.

## 3. DLVO Potential Calculation

Potential energy calculation between (a) two silica particles or (b) aluminium stub/plate and a silica particle was performed using the DLVO (Derjaguin–Landau–Verwey–Overbeek) theory, which is a well-known theory for describing the material interactions with the summation of the van der Waals potential (*V*_A_) and electrical double layer potential (*V*_R_) [7,8]. If the total potential energy (*V*_T_ = *V*_A_ + *V*_R_) is high and positive, particles repel each other; otherwise, particles attract each other. This is a straight-forward theory, which can explain particle coagulation/dispersion in many different colloidal systems, e.g., [9,10,11,12,13,14,15]. In our previous study that is relevant to the present study, the DLVO theory was also applied to investigate the particle–particle interactions in the system with the small quantity of water present in agglomeration processes [13]. The following paragraphs will introduce and explain the equations used for the potential energy calculation.

Equations used to calculate the potential energies between similar spherical particles [7,8]:

(1) $$V_{A}=-\frac{Aa}{12H}$$

(2) $$V_{R}\;=\;\frac{64\pi ankT\gamma^{2}\exp\left(-\kappa H\right)]}{\kappa^{2}}$$

(3) $$n\;=\;\;N_{A}C$$

(4) $$\kappa \;=\;{\left(\frac{8\pi nz^{2}e{\;}^{2}}{\varepsilon \varepsilon_{0}kT}\right)}^{\frac{1}{2}}$$

(5) $$\gamma \;=\;\frac{\exp\left(\frac{ze\zeta }{2kT}\right)-1}{\exp\left(\frac{ze\zeta }{2kT}\right)+1}$$

where *A* is the Hamaker (J) constant, a is the particle radius (nm), *H* is the inter-particle separation distance, n is the number concentration of ions (nm^−3^) defined in Equation (3), *N*_A_ is the Avogadro’s number ($6.022\;\times \;10^{23}\;\;{\mathrm{mol}}^{-1})$, *C* is the concentration of ions (mol/nm^3^), *k* is the Boltzmann constant ($1.38\;\times \;10^{-23}\;\;J/K)$, *T* is the absolute temperature (K), γ is the reduced surface potential (unitless), κ is the Debye–Huckel reciprocal length (nm^−1^) defined in Equation (4), ε is the dielectric constant of the medium, $\varepsilon_{0}$ is the permittivity of free space (C/Vnm), *z* is the ionic valence, *e* is the elementary charge (C), and ζ is the zeta potential (V). The zeta potential values of silica particles [16] and aluminium plate [17] were extracted from the literature and used for the present calculation.

Equations used to calculate the potential energy between plate and spherical particle interactions [18] (in our case, the interaction between the aluminium stub and silica particle):

(6) $$V_{A}\;=\;-\frac{A}{6}\left(\frac{a}{H}+\frac{a}{H+2a}\;+\;\ln\left(\frac{a}{H+2a}\right)\right)$$

(7) $$V_{R}\;=\;\frac{128\pi ankT\gamma_{s}\gamma_{p}\exp\left(-\kappa H\right)]}{\kappa^{2}}$$

where $\gamma_{s}$ and $\gamma_{p}$ are the reduced surface potential of the sphere and plate (unitless), respectively.

In this article, the calculated total potential energies were normalized by the thermal fluctuation energy (kT). For the dissimilar plate-particle systems, the Hamaker constant $A_{132}$ was calculated by using the following Equation [19]:

(8) $$A_{132}\;=\;\left(\sqrt{A_{11}}\;-\;\sqrt{A_{33}}\right)\left(\sqrt{A_{22}}\;-\;\sqrt{A_{33}}\right)$$

where $A_{11}$ is the Hamaker constant of particle 1 in vacuum, $A_{22}$ is the Hamaker constant of particle 2 in vacuum, and $A_{33}$ is the Hamaker constant of water in vacuum. These values were obtained from the literature [19,20].

## 4. Non-Destructive Imaging

**Figure 1:**
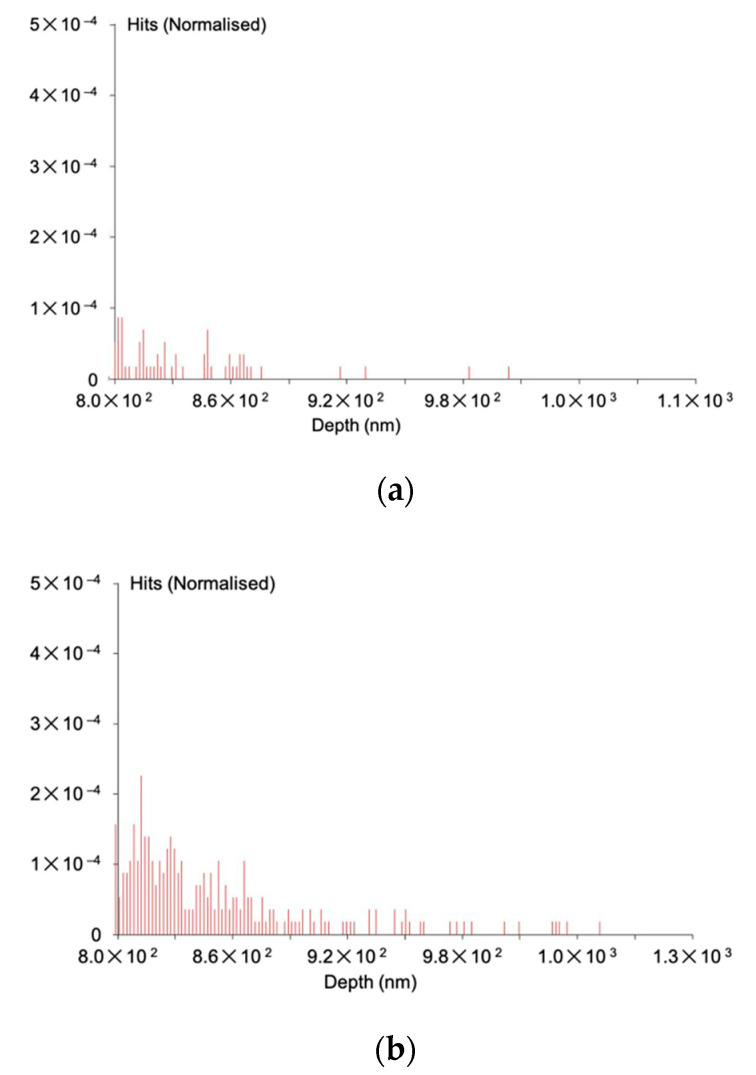
Backscattered electrons generated by the electron beam impacted on the nanoparticles at the acceleration voltages of (**a**) 8.1 and (**b**) 8.5 keV. These histograms are simulated by CASINO program [20].

As described in Section 1, the acceleration voltage of the electron beam in SEM was precisely controlled to achieve the corresponding penetration into the layer above and below the buried interface to be investigated. The detailed procedures of the non-destructive imaging we recently developed can be found in [3]. An electron flight simulator, CASINO [21], was used to calculate the number of BSEs to be generated in nanoparticles. For the cupric and silica nanoparticles investigated in this study, the simulations show that BSEs can be generated in the vicinity of the nanoparticle–substrate interfaces by introducing an electron beam accelerated at a series of voltages between 18 and 20 keV and between 8.1 and 8.5 keV, respectively. For the latter case for example, as shown in Figure 1a, BSEs are generated predominantly within the nanoparticles at 8.1 keV, while more BSEs are generated from both the nanoparticles and the substrate at 8.5 keV (see Figure 1b). After the lower-acceleration SEM image is subtracted by the higher-acceleration SEM images, buried interfaces can be revealed.

## 5. Nanoparticles Synthesised by X-ray Radiolysis

**Figure 2:**
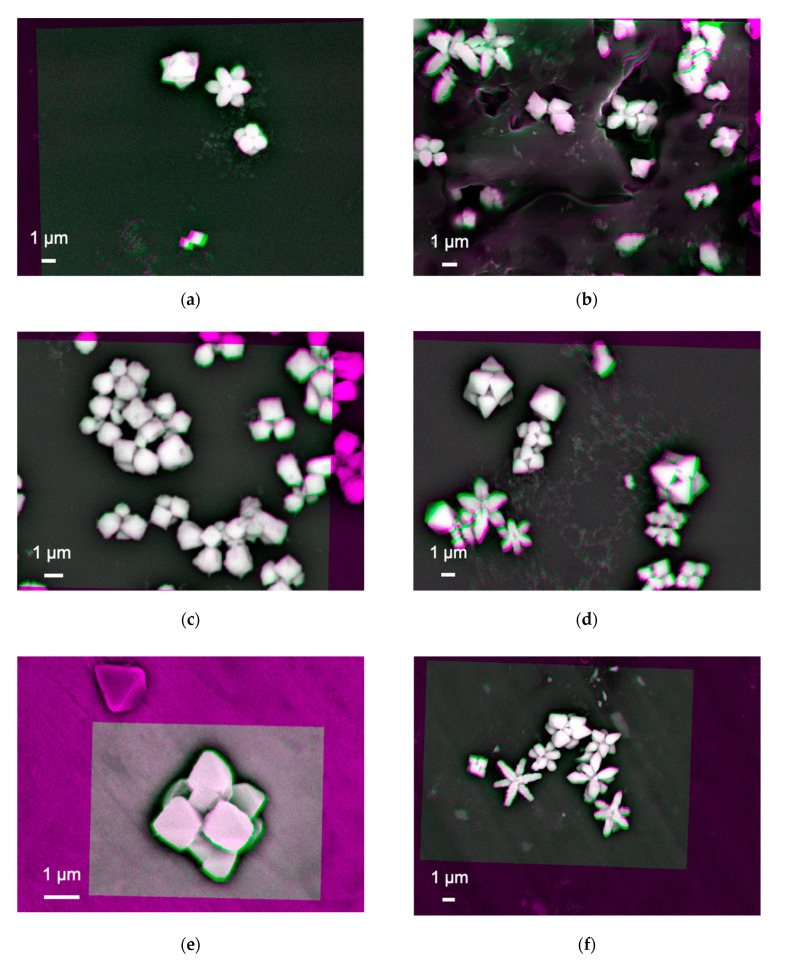

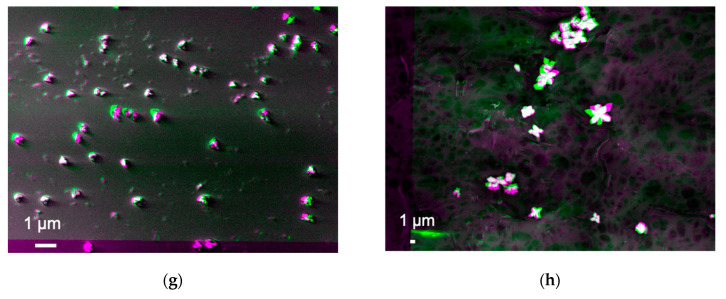
Processed images of the nanoparticle–substrate interfaces after subtracting two images taken at the acceleration voltages of 18 and 20 keV on the samples grown on (**a**) Si/SiO_2_, (**b**) *n*-Si(001), (**c**) *p*-Si, (**d**) *n*-Si(111), (**e**) Ni, (**f**) Al, (**g**) 128° Y-cut LiNbO_3_ and (**h**) PTFE substrates.

The nanoparticles, #1 and 6, were imaged as shown in Figure 2a–h, respectively. These images were produced after subtracting two SEM images, which have been taken using different acceleration voltages of 18 and 20 keV. These images need to be aligned, which was carried out by adjusting the positions of the nanoparticles within the orange box shown in each image. The colour changes from magenta to green indicate that there are defects or vacancies within the subtracted image. The magenta colour shown at the edge of the particles in these images is due to its spherical shape as there is no intimate contact between the edge of the particles and the substrate. In addition, the bright and dark regions represent the number of BSEs generated to be more and less, respectively.

Figure 2a shows almost white and bright contrast at the nanoparticle–substrate interfaces, indicating that the interfaces are uniformly formed to generate a sufficient number of BSEs. Some arm-shaped regions appeared in magenta and green colours, indicating BSEs are generated above and below the interfaces, respectively. The magenta- and green-coloured features may indicate that the arm regions of the nanoparticles can be detached by voids. This may suggest that these arms can be formed once the main body of the nanoparticle (the middle region) is formed.

Figure 2b shows the nanoparticles synthesised on a *n*-doped Si(001) substrate with a sheet resistance of 1~10 Ω⋅cm. The size of the nanoparticles is found to be slightly randomised but maintains elongated shapes as seen in Figure 2a. The nanoparticle–substrate interfaces show broad distributions of contrast. This indicates that some nanoparticles with white bright interfaces are formed in the same manner with those synthesised on the Si/SiO_2_. However, additional nanoparticles may have moved to form clusters, possibly due to the conductivity of the substrate.

By replacing the substrate with *p*-doped Si(001) with a sheet resistance of 1~20 Ω⋅cm, the clustering of the nanoparticles is slightly suppressed by increasing the separation between the nanoparticles as shown in Figure 2c. The shape also becomes square like. The interfaces stay uniform. Their elongation is recovered by synthesising them on *n*-doped Si(111) as shown in Figure 2d, promoting triangular-shaped particles with closer clustering like those on *n*-Si(001).

On the other hand, the nanoparticles synthesised on the metallic Ni substrate show white bright contrast with magenta colour only without any arms as shown in Figure 2e. The shape and size of the nanoparticles are found to be almost cubic with three-fold symmetry as observed for the Si substrates as described above. Similar structures with more elongation are observed for the Al substrate as shown in Figure 2f. Randomly formed nanoparticles are observed for those synthesised on a 128° Y-cut LiNbO_3_ substrate (see Figure 2g). In addition, some distorted particles are observed as immobilised on the LiNbO_3_ substrate. They may be due to the chemical interactions with Cu(COOCH_3_)_2_ and difference in crystallinity between LiNbO_3_ and cuprates. They become elongated on Al and polytetrafluoroethylene (PTFE) substrates. These results indicate that secondary electrons from the substrates by the X-ray introduction may contribute to the nucleation, growth, and aggregation of nanoparticles. It should be noted that all these samples maintain consistent interfaces.

**Figure 3:**
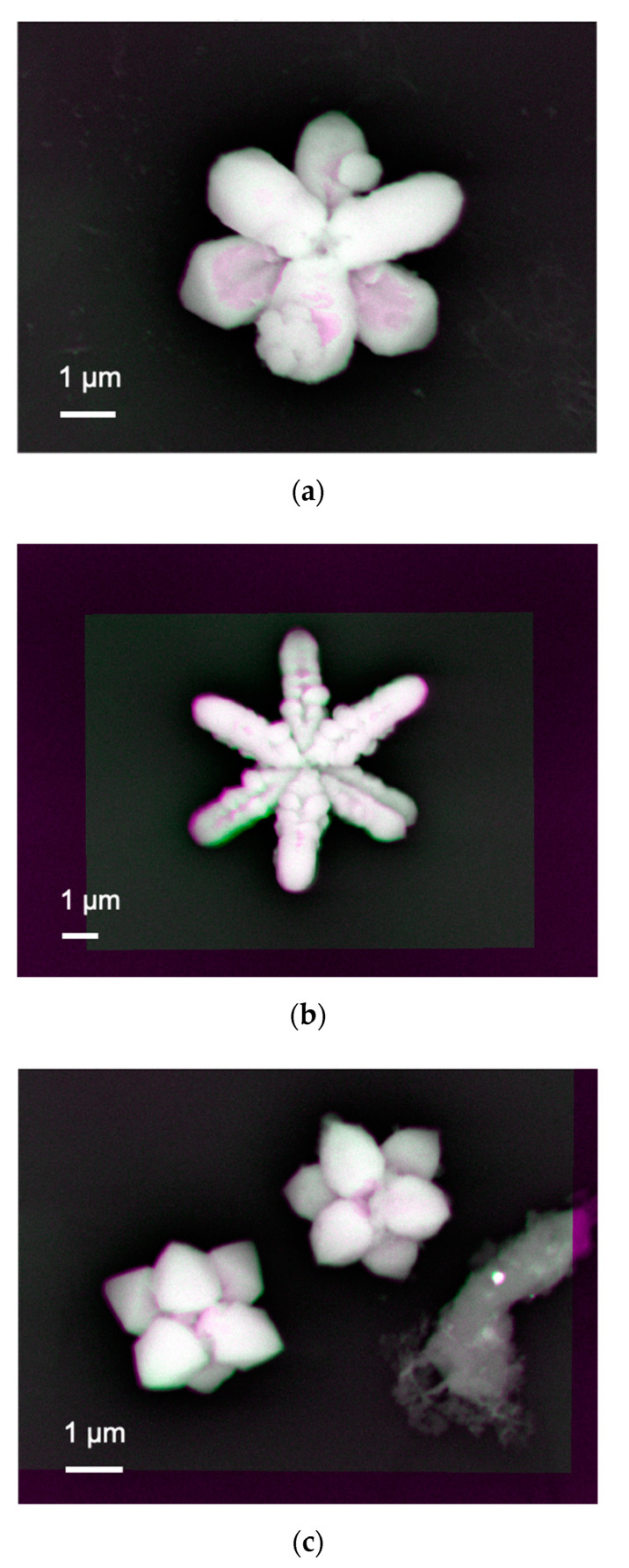
Processed images of the nanoparticle–substrate interfaces after subtracting two images 18 and 20 keV on the samples grown on Si/SiO_2_ at pH = (**a**) 8, (**b**) 9, and (**c**) 7.

Since prominent elongated arm-like features were obtained for those synthesised on Si/SiO_2_ substrates, we further imaged nanoparticles synthesised under a series of pH between 7 and 9. Figure 3a shows almost white bright contrast at the nanoparticle–substrate interfaces for the Y-shaped nanoparticle, confirming that the interface is uniformly formed to generate a sufficient number of BSEs. There are some minor distributions in colour, where some voids exist at the interface. The inverse Y-shaped nanoparticle is found to be formed on the Y-shaped one as the inverse Y-shaped one has darker contrast, indicating the corresponding interface generates less BSEs, i.e., possible presence of voids.

A similar interfacial structure is observed by increasing pH to 9 as shown in Figure 3b. The contrast between the Y-shaped and inverse Y-shaped nanoparticles become weaker, meaning the interface for the latter one reduces the voids, i.e., the formation of a uniform interface. At the same time, each arm becomes more granular than that for the sample synthesised at pH = 8. This means the nanoparticle with the two overlapping Y-shapes is formed by clustering small circular particles to grow along six crystalline facets.

For pH = 7, the growth along the six facets becomes weaker to form less elongated arms as seen in Figure 3c. Again, the bottom Y-shaped nanoparticles have stronger adhesion to the substrate than the inverse Y-shaped one. By increasing pH to 11, the nanoparticles become almost like a sphere by attaching only the bottom centre of them. These results suggest that these cuprates are formed from triangular seed crystals, followed by preferred facet growth along the three directions. These cuprates grow in a Y-shape, whose arm length depends on the substrate and pH, which control the mobility of the seed crystals. By rotating 60° the Y-shaped cuprates to overlap each other, two of them can form a hexagonal structure [5]. These results shown here indicate that the composition and crystal shape of the synthesised and immobilised cupric nanoparticles are dependent on the conductivity of the substrates and pH of the liquid solutions. The formation of synthesised crystal can be modified when the relative order of the surface energies is altered or when the crystal growth along certain directions is selectively hindered. These results suggest that the selection of surface crystal structures and the electronic states of substrates play a dominant role in controlling the synthesis of nanoparticles.

## 6. Nanoparticles Synthesised by the Sol-Gel Method and Their Aqueous Suspensions

**Figure 4:**
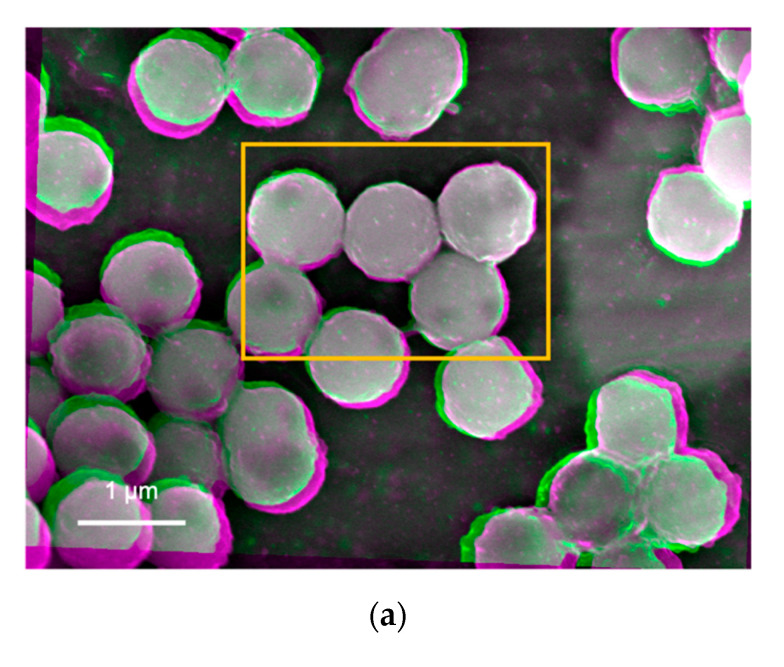

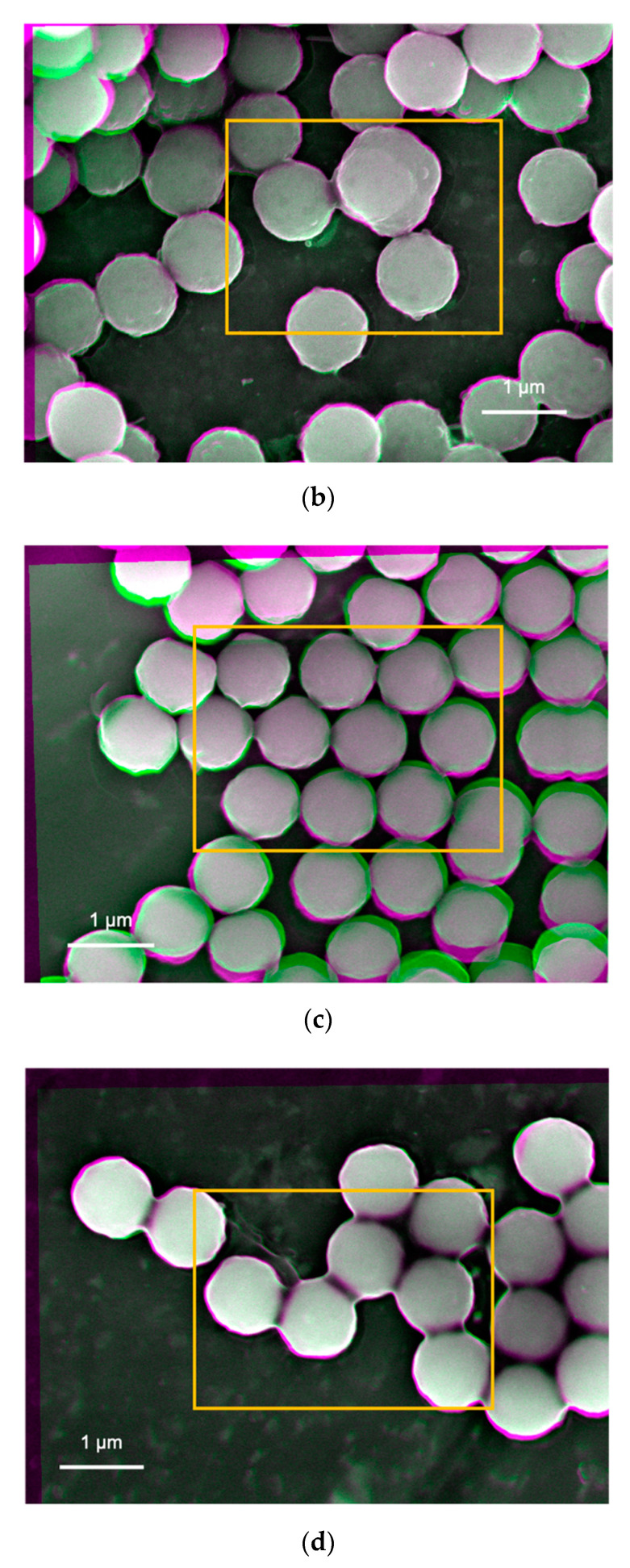
Processed images of the nanoparticle–substrate interfaces after subtracting two images taken at the acceleration voltages of 8.5 and 8.1 keV on the samples of Tug (**a**) 2, (**b**) 3, (**c**) 5, and (**d**) 6.

**Figure 5:**
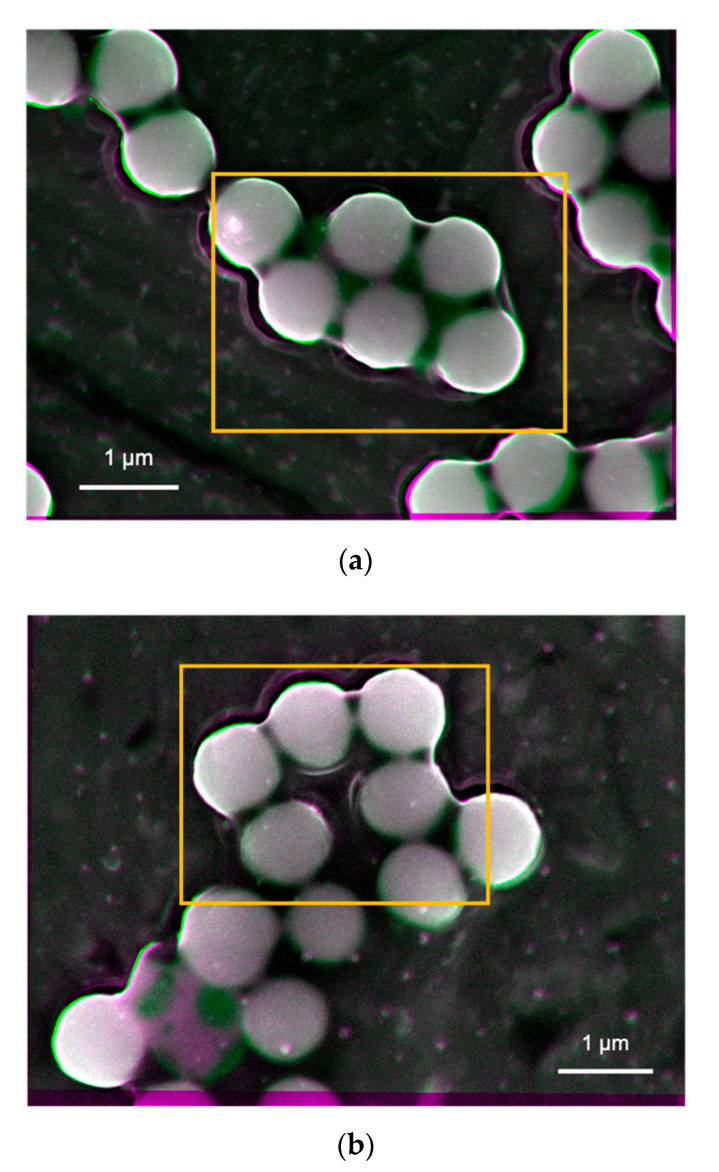
Processed images of the nanoparticle–substrate interfaces after subtracting two images taken at the acceleration voltages of 8.5 and 8.1 keV on the Tug 3 sample with different areas (**a**,**b**).

**Figure 6:**
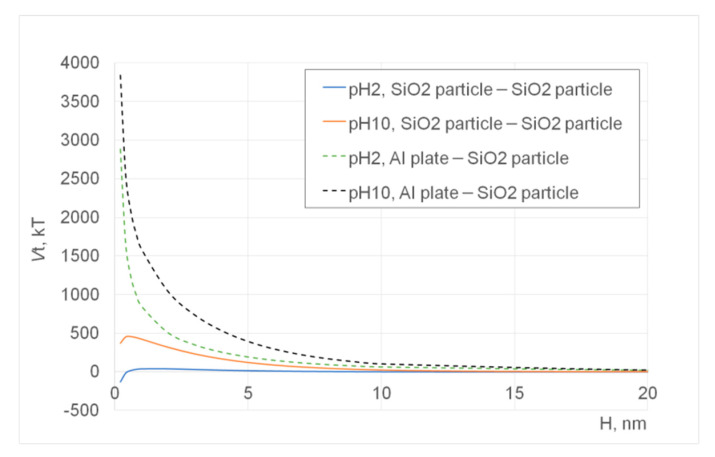
DLVO total potential energies between silica–silica particles with the radius of 280 nm as well as between the Al stub plate and silica particles at pH 2 or 10 and 1 × 10^−2^ M KNO_3_ aqueous solution at 25 °C, as a function of interparticle separation distance. The unit of the potential, *V*t is the thermal energy, *k*_B_*T* [J].

Suspensions prepared by the nanoparticles created by the sol-gel method were imaged as shown in Figure 4a–d, respectively, while some additional images were taken for a sample (i.e., Tug 3) as shown in Figure 5. These images were again produced after subtracting two SEM images, which were taken using different acceleration voltages of 8.1 and 8.5 keV. On the other hand, the results of the DLVO potential calculation are shown in Figure 6.

In the processed images shown in Figure 4 and Figure 5, in general, the edges of these particles show bright/white colour, indicating that they generate more BSEs as compared with the other interfaces. This suggests that the particles are pinned by these edges due to the capillary force that is often expressed by the neck shape structure between a particle and a plate in the presence of a small amount of water [22,23,24,25]. On the other hand, some edges are shown in green or magenta colour, indicating that they are detached from the substrate due to repulsive force that can be explained by the electrostatic interactions between particles and/or particle and the substrate/stub.

In Figure 4a, some nanoparticles contain grey spots (less BSEs), indicating there are some defects or vacancies formed at the nanoparticle, substrate, or their interface. Minor bright edges are observed while other edges in green or magenta are also seen in Tug 2 (Figure 4a), indicating that the nanoparticles are deposited on the substrate with some instability due to electrostatic repulsion among the highly charged silica particles at pH 10 (Tug 2), as the repulsive DLVO potential interaction among them is shown in Figure 6 (pH 10 SiO_2_ particle–SiO_2_ particle; pH 10 Al plate–SiO_2_ particle). It is more noticeable with Tug 2 prepared at pH 10 (Figure 4a) than Tug 5 at pH 2 (Figure 4c), and this agrees with the DLVO potential calculation shown in Figure 6 (pH 10 SiO_2_ particle–SiO_2_ particle vs. pH 2 SiO_2_ particle–SiO_2_ particle) explaining the repulsive interaction at pH 10 while the attractive interaction at pH 2 between SiO_2_ particles. Here, it is worth mentioning that the capillary force can be stronger than the attractive DLVO forces [26] (mainly van der Waals force in our case) in order to keep those particles on the substrate/stub while the repulsive DLVO forces (electrostatic force in our case) can influence the stability of particle deposition on the substrate/stub. Similarly, Figure 4c shows almost uniform interfaces at the middle of the nanoparticles but with some edge defects as shown in the green colour. Tug 5 (Figure 4c, pH 2) has a more flat structure in comparison with Tug 2 (Figure 4a, pH 10) that forms multilayer disposition at the same particle concentration of 0.1 vol.%, indicating more stable adhesion between the silica particles and substrate, as the attractive DLVO potential interaction among them at pH 2 shows in Figure 6 (pH 2 SiO_2_ particle-SiO_2_ particle).

Figure 4b shows similar interfacial contrast but with darker regions at the edges of the nanoparticles whose suspension was prepared at pH 10 and 0.01 vol.% silica. Furthermore, some interparticle spots in green colour are observed. This indicates that the silica nanoparticles do not have a perfect spherical shape or have repulsive interactions to separate the nanoparticles from each other. On the other hand, Figure 4d shows no interparticle spots in the green colour, indicating attractive interactions between particles as agreed with the DLVO potential calculation (Figure 6, pH 2 SiO_2_ particle–SiO_2_ particle). The more uniform colour regions are observed in the Tug 3 (Figure 4b) and Tug 6 (Figure 4d) prepared at 0.01 vol.% silica, where there is a higher amount of moisture content than 0.1 vol.% (Tug 2, Figure 4a and Tug 5, Figure 4c), and it indicated that silica nanoparticles are firmly deposited on the stub by the capillary force with the residual water.

In terms of the interactions between the aluminium stub and silica particles, they are more repulsive at pH 10 and 0.1 vol.% silica particles (Tug 2) than at pH 2 and 0.1 vol.% silica particles (Tug 5), as shown in Figure 4a,c and agreed with the highly positive DLVO potential energies in the former sample condition (Figure 6, pH 10 vs. pH 2) and literature [16]. It can be also explained by the isoelectric point (IEP) of silica, which is around pH 2, where silica particles can coagulate while pH 10 is where the solution pH is far away from IEP and thus silica particles repel each other [16]. Comparing between 0.1 vol.% (Tug 2, pH 10) and 0.01 vol.% (Tug 3, pH 10), the former solid concentration provides more repulsive–instable interactions. It can be explained by the presence of a higher number of particles that are deposited on the aluminium stub with the repulsive nature of interactions.

SEM images were also taken in the other three different areas for each sample to confirm the representativity of the observed images shown in Figure 5 that are the processed images of Tug 3 (pH 10, 0.01 vol.%) as an example. No apparent difference is observed for all the four samples, apart from a minor difference in the particle orientation for the Tug 3. It can be explained by a non-even particle distribution as shown in Figure 5.

## 7. Summary

By using our non-destructive imaging method, we imaged nanoparticles synthesised by X-ray radiolysis and the sol-gel method. The X-ray radiolysis is found to initiate the formation of a triangular seed crystal, followed by growth along three facet directions. The sol-gel method, on the other hand, forms spherical nanoparticles, which are pinned to the substrate at the interface and clustered randomly. These crystallisation, deposition, and aggregation processes can be controlled by the substrates, pH, and density as expected and agreed with colloidal DLVO theory. Our imaging method can offer an ideal feedback to achieve precise control of the synthesis processes.

## Figures and Tables

**Table 1:** List of nanoparticles synthesised by X-ray radiolysis.

Samples	Cu(COOCH_3_)_2_ Volume	Additive Solution and Volume	Substrates
#1	200 µL	Methanol 1 µL	Si/SiO_2_
#2	200 µL	Methanol 1 µL	*n*-Si(001)
#3	200 µL	Methanol 1 µL	*p*-Si(001)
#4	200 µL	Methanol 1 µL	*n*-Si(111)
#5	200 µL	Methanol 1 µL	Ni
#6	200 µL	Methanol 1 µL	Al
#7	200 µL	Methanol 1 µL	LiNbO_3_
#8	200 µL	Methanol 1 µL	PTFE
#9	900 µL	Methanol 5 µL + NH_3_ 100 µL (pH = 8)	Si/SiO_2_
#10	900 µL	Methanol 5 µL + NH_3_ 200 µL (pH = 9)	Si/SiO_2_
#11	950 µL	Methanol 5 µL + NH_3_ 50 µL (pH = 7)	Si/SiO_2_
#12	500 µL	Methanol 5 µL + NH_3_ 500 µL (pH = 11)	Si/SiO_2_

**Table 2:** List of the silica nanoparticle suspensions studied.

Samples	Preparation Methods
Tug 2	Silica 0.1 vol.%, 1 × 10^−2^ M KNO_3_, pH10
Tug 3	Silica 0.01 vol.%, 1 × 10^−2^ M KNO_3_, pH10
Tug 5	Silica 0.1 vol.%, 1 × 10^−2^ M KNO_3_, pH2
Tug 6	Silica 0.01 vol.%, 1 × 10^−2^ M KNO_3_, pH2

## Data Availability

Data is contained within the article and available on request with following the guideline set by the University of York (UK).
